# Self-uncertainty and conservatism during the COVID-19 pandemic predict perceived threat and engagement in risky social behaviors

**DOI:** 10.1177/13684302231180525

**Published:** 2023-07-07

**Authors:** Lily Syfers, Alexandria Jaurique, Benjamin Anjierwerden, Sara E. Burke, Justin D. Hackett, David E. Rast, Amber M. Gaffney

**Affiliations:** 1 University of Alberta, Canada; 2 Syracuse University, USA; 3 Cal Poly Humboldt, USA; 4 Pennsylvania Western University, California, USA

**Keywords:** COVID-19, realistic threat, self-uncertainty, symbolic threat

## Abstract

Two studies (*N* = 676) highlight the nuanced relationship between conservatism and adherence to COVID-19 policy and recommendations intended to slow the spread of the pandemic in the United States. Study 1 provided evidence that conservative Americans who felt uncertain about themselves and the future experienced elevated levels of symbolic threat (attacks to sociopolitical identity; e.g., the pandemic threatening American democracy) and realistic threat (concrete attacks to material resources or well-being; e.g., the pandemic threatening physical health) in comparison to their more certain counterparts. In Study 2, the association between this form of uncertainty and frequency of risky social behaviors (behaviors that increase the risk of virus transmission) was partially mediated by threat perception for Americans both low and high in conservatism. We discuss findings as an integration of the motivated social cognition framework and uncertainty-identity theory. While self-uncertainty was more associated with greater overall COVID-19 threat perception for Americans high (vs. low) in conservatism, threat perception and frequency of risky social behaviors were associated with self-uncertainty in a manner that is consistent with prevailing liberal and conservative norms.

Political conservatism is underpinned by motivations to manage and reduce threat and uncertainty, which manifests in conservatives displaying greater sensitivity to a variety of threats thanliberals (Jost & Amodio, 2012; Jost et al., 2003, 2007). The COVID-19 pandemic is unique in that American conservatives are less likely than liberals to perceive the virus as a threat to public health (Tyson, 2020), and they tend to experience lower anxiety and less perceived susceptibility to the virus than their liberal counterparts (Calvillo et al., 2020; Kempthorne & Terrizzi, 2021; Kerr et al., 2021). Moreover, conservatives have engaged in significantly less social distancing than liberals throughout the pandemic, which would have provided some protection from the virus (Enten, 2021; Gollwitzer et al., 2020; Leventhal et al., 2021). In the beginning of the pandemic, counties with a 2016 majority vote for Donald Trump (the Republican presidential nominee) tended to show higher infection rates than counties with a 2016 majority vote for Hillary Clinton (the Democratic presidential nominee). The partisan pattern in infection rates and deaths became more extreme after vaccines became available—in October 2021, the death rate in Republican counties was 3 times that of Democratic counties (Leonhardt, 2021). COVID-19 is an undeniably politicized issue; therefore, it is crucial to understand how ideological group norms inform threat perception and behavior. We conceptualize conservatism as a bipolar construct where low scores (below the midpoint) on the Conservatism Scale indicate liberalism, and high scores (above the midpoint) indicate conservatism. Thus, people who are low in conservatism are aligned with liberal ideology, and those high in conservatism are aligned with conservative ideology. The present work integrates uncertainty-identity theory (Hogg, 2007, 2021; Hogg & Gøtzsche-Astrup, 2021) and the motivated social cognition framework (Jost & Amodio, 2012; Jost et al., 2003, 2007) to examine whether self-conceptual uncertainty is a stronger predictor of threat perceptions for Americans high (compared to low) in conservatism. This work further examines whether self-uncertainty is indirectly associated with frequency of risky social behaviors (behaviors conducive to spreading COVID-19; e.g., going to crowded events) through perceived realistic and symbolic threat, and whether this relationship is stronger for Americans high (rather than low) in conservatism. The goal of this work is to use a framework that includes uncertainty-identity theory (Hogg, 2007, 2021; Hogg & Gøtzsche-Astrup, 2021) and motivated social cognition (Jost & Amodio, 2012; Jost et al., 2003, 2007, 2008) as predictors of both COVID-19 perceived threats and related behaviors. Given the times of data collection (Summer 2020 and Spring 2021) and our research goals, we employed correlational designs; thus, the current work cannot be used to establish causation.

## Motivational Underpinnings of Political Ideology

Those with strong dispositional or situational motivations to reduce perceived threat and uncertainty tend to gravitate toward conservatism, presumably because conservative ideology prioritizes familiarity and stability through maintenance of the status quo (Jost et al., 2003). Jost and colleagues distinguish conservative and liberal ideologies by (a) resistance versus advocacy of change, and (b) acceptance versus rejection of inequality. Research has consistently linked dispositional characteristics associated with higher needs for uncertainty management (e.g., intolerance of ambiguity, uncertainty avoidance, need for cognitive closure, lack of openness to experience), as well as basic cognitive and neural processes associated with threat sensitivity (e.g., belief in a dangerous world, death anxiety), to conservatism (Amodio et al., 2007; Jost & Amodio, 2012; Jost et al., 2003, 2007, 2008; Oxley et al., 2008). Uncertainty avoidance and threat sensitivity seem to be independent clusters of constructs that both distinctly predict a greater attraction to political conservatism (Jost et al., 2007). Situational factors that increase uncertainty and threat can also increase the attractiveness of political conservatism. When societal systems are threatened, the prospect of social change is often risky and uncertain. In response to situationally increased levels of uncertainty and threat, even relatively liberal college students can gravitate toward aspects of conservative ideology because it is better suited to the mitigation of uncertainty and threat than liberalism’s advocacy of change and equality (which itself often requires large-scale change to achieve; see Jost & Napier, 2011; Jost et al., 2003, 2007; van der Toorn et al., 2017). However, the operationalizations of uncertainty and threat used in research impact whether the expected distinctions between motivations underpinning liberalism and conservatism are observed. For example, a review of the conservatism literature highlights clearer differences in threat sensitivity between liberals and conservatives in response to physical, but not symbolic (meaning and value of identity), threats (Crawford, 2017); yet, Bakker et al. (2020) did not find any significant differences in physiological reactions to physical threats (e.g., picture of a spider on a person’s face, picture of a person with a bloody face). Although it appears that conservatives may be more averse to some types of uncertainty and to threat in general, they also report greater ambivalence toward several kinds of attitude objects compared to liberals, which arguably contradicts the tight theoretical link between conservatism and clusters of uncertainty intolerance constructs (Newman & Sargent, 2021; Sargent & Newman, 2021). The empirical work on the relationship between threats, uncertainty, and conservatism has several broad operationalizations of threat and uncertainty. The present research examines a particular type of uncertainty—self-uncertainty—and two distinct types of threat—realistic and symbolic—in the situational context of the COVID-19 pandemic, which created large system, societal, and personal instability surrounding safety, the economy, livelihoods, and identity.

Uncertainty-identity theory (Hogg, 2007, 2020, 2021, 2022) proposes that external events can cause people to feel adverse uncertainty relating to their self-concept, which people are motivated to reduce by attending to identity-relevant information that is derived from existing or new memberships in social groups. During the COVID-19 pandemic, virtually all aspects of daily life were suddenly disrupted. For some people, their entire life trajectory shifted after losing their jobs or being delayed in achieving their life goals. Many people experienced social isolation and interruption in daily routines and meaningful activities. These disruptions were associated with distressing uncertainties (e.g., Salah et al., 2022; Zavras, 2021). Because uncertainties and threats were so prevalent during the COVID-19 pandemic, it is challenging to tease apart potential causal relationships.

The relationship between self-uncertainty and threat has previously been studied in the context of identification with extremist groups (Hogg et al., 2010, 2014, 2021), intergroup threat (Niedbala & Hohman, 2019; Rast et al., 2017), and self-representation threats (Kuljian et al., 2023). In the context of COVID-19, Chen et al. (2022) demonstrated that perceptions that the pandemic threatens physical health, financial well-being, and day-to-day life (realistic threats) partially mediated the positive relationship between American identity certainty and support for restrictive health behaviors. Perceptions that the pandemic threatened the meaning and value of American identity (symbolic threats) partially mediated the positive relationship between American identity certainty and COVID-19 conspiracy beliefs. Certainty about the normative content of American identity may have helped people to decide which aspects of the pandemic were threatening and how to respond to those threats.

Due to the motivational nature of self-uncertainty, the reverse should also be true. Self-uncertainty motivates people to turn toward accessible and salient group identities to reduce the uncertainty by conforming to the normative content of the group identity (Hogg, 2007, 2020, 2021, 2022). In the case of COVID-19, attitudes, beliefs, and behaviors quickly polarized along partisan lines (Calvillo et al., 2020; Enten, 2021; Gollwitzer et al., 2020; Kempthorne & Terrizzi, 2021; Kerr et al., 2021; Leventhal et al., 2021), which indicates that the motivational nature of self-uncertainty should prompt people to tie their beliefs and behaviors to their ideological identities. For this reason, we propose self-uncertainty as the independent variable in our theoretical model. Of course, the unprecedented crisis nature of the pandemic could incite self-uncertainty, symbolic threats, and realistic threats all at the same time. However, the motivational nature of self-uncertainty could guide people to conform to group norms as the lens to filter and make sense of information. In this way, although self-uncertainty and multiple types of threat can arise simultaneously, self-uncertainty could motivate people to strengthen ties to an accessible social identity (i.e., a political identity with a clearly structured ideology for resolving the uncertainty), which could eventually lead them to adjust their perceptions of threat to match those prescribed by their ideological group norms (Hogg et al., 2011; Mullin & Hogg, 1999; Smith & Hogg, 2008).

Both the motivated social cognition perspective (Jost & Amodio, 2012; Jost et al., 2003, 2007, 2008) and uncertainty-identity theory (Hogg, 2007, 2020, 2021, 2022) provide analyses of the uncertainty-reducing function of political ideologies. However, uncertainty-identity theory focuses specifically on self-conceptual uncertainty being a motivational underpinning of identification with social groups in general, whilst the motivated cognition perspective proposes that liberal and conservative ideologies have differing capacity to manage situational or chronic needs to reduce threat and uncertainty. In addition, the motivated social cognition framework has examined the relationship between conservatism and numerous constructs relating to uncertainty intolerance (e.g., need for order, need for cognitive closure, intolerance of ambiguity, lack of openness to experience; Federico et al., 2012; Jost et al., 2007) and various sources of uncertainty (e.g., system instability, Jost et al., 2007; death anxiety, Jost et al., 2003, 2008). While the motivated social cognition framework and uncertainty-identity theory make unique predictions about ideological beliefs and behaviors, this paper presents an integration of the two theories to predict threat perception and virus spreading behaviors in the highly uncertain and threatening COVID-19 pandemic context. We propose that the theoretical relationship between uncertainty and threat depends on the type of uncertainty and the type of threat. Specifically, we propose that self-uncertainty predicts an increase in threat perception that is prescribed by group norms. However, considering the motivational underpinnings of political ideology, the strength and direction of the relationship between self-uncertainty and threat perception should depend on the degree to which an individual is politically conservative.

## Realistic and Symbolic COVID-19 Threats

Given that the pandemic was an unprecedented crisis for the global community, many people had no idea how to understand or react to the crisis or to their changing lives. Responses to the pandemic were quickly polarized across U.S. political party lines, with conservative politicians and media largely de-emphasizing the threat of viral infection and minimizing the importance of public health prevention measures. In contrast, liberal politicians and media mainly emphasized the risk of infection and the importance of social distancing and prevention measures (e.g., Canes-Wrone et al., 2022; Flores et al., 2022; Hegland et al., 2022; Ritter, 2020; Rothwell & Desai, 2020). Early in the pandemic, a clear pattern emerged in which right-leaning ideology was associated with less social distancing, more COVID-19 infections, and more deaths than left-leaning ideology (Leonhardt, 2021a, 2021b).

We define COVID-19 threat using Kachanoff et al.’s (2021) categorization of realistic and symbolic COVID-19 threat dimensions (see Stephan & Stephan, 2000). COVID-19 realistic threats involve tangible threats to material possessions and physical wellbeing, including physical health, financial health, the economy, and the familiarity and structure of daily life. COVID-19 symbolic threats attack core aspects of American identity and the meaning of being American, including American values and traditions, American democracy, and the maintenance of law and order. Kachanoff et al. found that realistic pandemic threat was negatively related to conservatism, whereas symbolic pandemic threat was weakly and inconsistently positively associated with conservatism. Liberal values emphasize universalism (moral obligation to a broad social network), egalitarianism, and government responsibility to helping citizens, whereas conservative values emphasize parochialism (moral obligation to a tight and less permeable social network), personal autonomy, personal responsibility, and opposition to government intervention (Collins et al., 2021; Jost, 2017; Jost et al., 2003; van Kessel & Quinn, 2020; Waytz et al., 2019). These values are reflected in the propensity of liberals to be concerned about COVID-19’s threat to physical wellbeing and public health (Kempthorne & Terrizzi, 2021; Kerr et al., 2021), and in their higher engagement, compared to conservatives, in public health behaviors aimed to mitigate that threat (Clinton et al., 2021; Gollwitzer et al., 2020; Latkin et al., 2022). Conservative values involving concerns over individual freedoms, liberty, and opposition to government intervention laid the groundwork for antimask, antilockdown, and antisocial distancing attitudes, which may be rooted in symbolic threat to core conservative American values (Collins et al., 2021; Jost, 2017; Jost et al., 2003; van Kessel & Quinn, 2020; Waytz et al., 2019). In the context of the COVID-19 pandemic, conservative norms encourage people to perceive greater symbolic than realistic threat (e.g., freedom threat vs. health threat), whereas liberal norms encourage people to perceive greater realistic than symbolic threat.

## Proposed Theoretical Model

Given the motivational underpinnings of conservative and liberal ideologies theorized by the motivated social cognition perspective, a reasonable conjecture is that U.S. conservatives, overall, have a greater base level motivation to avoid uncertainty and manage certain types of threat than liberals do (e.g., Amodio et al., 2007; Jost & Amodio, 2012; Jost et al., 2003, 2007, 2008). With the addition of a self-conceptual uncertainty reduction motive, conservatives should look toward their conservative group identity to inform their perception of the world (Hogg, 2007, 2020, 2021, 2022). As stated before, conservatives normatively focus more on symbolic than realistic COVID-19 threats (Collins et al., 2021; Jost, 2017; Jost et al., 2003; Kempthorne & Terrizzi, 2021; Kerr et al., 2021; van Kessel & Quinn, 2020; Waytz et al., 2019). Thus, the uncertainty-identity theory predicts that self-uncertainty will strengthen perceived symbolic threat more than realistic threat for conservatives, and realistic threat more than symbolic threat for liberals. As there is evidence that conservatives are more responsive to some threats and types of uncertainty than liberals (e.g., Jost, 2017; Jost et al., 2003, 2008), we cannot rule out that self-conceptual uncertainty, in tandem with greater base-level motivations to manage threat and uncertainty, has a broader association with threat perception for people high (vs. low) in conservatism. We predict that for people low in conservatism, self-uncertainty should have a stronger relationship with realistic than symbolic threat, and that the relationship between self-uncertainty and symbolic threat perceptions should be stronger for people high (vs. low) in conservatism.

## Alternative Models

To our knowledge, there is no published empirical data evaluating whether the effect of self-conceptual uncertainty on perceptions and behaviors varies as a function of the unique motivational underpinnings of conservatism and liberalism. Because of this lacuna, and the correlational nature of the data presented in this paper, there are two alternative theoretical models that must be considered before moving forward. The first plausible alternative model is one in which perceived realistic and symbolic threats are precursors to self-uncertainty more for those high, compared to low, in conservatism. Self-uncertainty motivates conformity to group norms, which shape and dictate the environmental information people consider important and correct (Choi & Hogg, 2020; Grant & Hogg, 2012; Grieve & Hogg, 1999; Hogg & Mahajan, 2018; Hogg, 2007; Hogg et al., 1998, 2010; Hohman & Hogg, 2015; Mullin & Hogg, 1999). Thus, regardless of whether self-uncertainty and realistic and symbolic threat perceptions activate simultaneously, self-uncertainty will eventually guide people toward focusing on the specific threat type(s) that is/are prescribed by their ideological identity.

The second plausible alternative model is that self-uncertainty should maximize perceptions of threat types that are prescribed by group norms, and minimize those that are inconsistent with group norms. In this case, self-uncertainty might predict increased symbolic and realistic threat perceptions for those high in conservatism, and might increase realistic threat perceptions while decreasing symbolic threat perceptions for those low in conservatism. Uncertainty-identity theory draws on the principles of self-categorization theory, which stipulate that ingroup norms are defined in relation to a contextually relevant outgroup (Turner et al., 1987). In the context of COVID-19, partisan intergroup differences became polarized, with liberal norms defined in contrast to conservative norms and vice versa (see Abrams et al., 2021). For example, perceived political polarization predicted the most pronounced differences in public health measures support and vaccine compliance (Cole et al., 2022; Dolman et al., 2023; Kerr et al., 2021), and mask wearing was symbolic of people’s partisan social identities (e.g., Powdthavee et al., 2021; Shin et al., 2022). If the group norms explicitly reject or discourage a certain belief, attitude, or behavior, then uncertainty-identity theory predicts that self-uncertainty should selectively minimize cognition and behaviors that are antinormative, as part of the social influence process and conformity to group norms. This theoretical proposition is intriguing and consistent with the core tenets of self-categorization theory and uncertainty-identity theory (Hogg, 2007, 2020, 2021, 2022; Turner et al., 1987). To test this proposition, an experiment may examine whether self-categorization into a political identity maximizes ingroup normative threat perception, while threat perception attributed to outgroup norms is minimized. As the data are correlational, we did not isolate political identity and cannot account for various other social identities and norms at play. Considering these methodological factors, we expect self-uncertainty to be associated with higher levels of realistic threat perception, compared to symbolic threat perception, for people low in conservatism, but we do not have a specific directional hypothesis for the relationship between self-uncertainty and symbolic threat perception.

There are also, of course, plausible models that do not stem from any causal relationships among the measured variables. For example, an unmeasured third variable, such as susceptibility to misinformation, may correlate with conservatism and cause people to report experiencing some forms of uncertainty and threat. Our design does not permit us to rule out such explanations.

## Risky Social Behaviors

**Figure 1.**
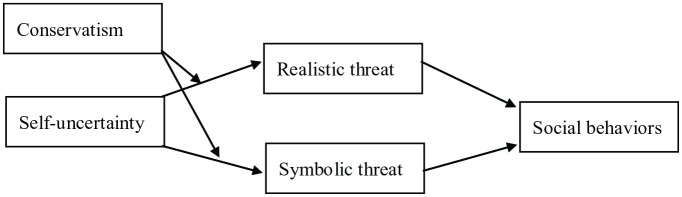
Conceptual parallel moderated mediation model in Study 2.

The key implication of the relationship between self-uncertainty and group-based threat perception is that if political ideology dictates threat perception, then ideological norms should also dictate the appropriate response to mitigate the perceived threats. Risky social behaviors are behaviors that increase the risk of spreading COVID-19. These include behaviors like socializing without masks or social distancing, going to crowded events, and going out to eat (Infectious Diseases Society of America, n.d.; Kim & Crimmins, 2020; Salimi et al., 2020; Texas Medical Association, 2020). Drawing from previous literature, we expect that perceived realistic threat should be negatively associated with engagement in risky social behaviors (Chen et al., 2022; Kachanoff et al., 2021), and symbolic threat should be positively associated with engagement in risky social behaviors (Enten, 2021; Gollwitzer et al., 2020; Kachanoff et al., 2021; Leventhal et al., 2021). Realistic and symbolic COVID-19 threat perceptions are moderately positively correlated (Chen et al., 2022; Kachanoff et al., 2021), indicating that people who perceive high levels of both types of threat may have competing pressures motivating engagement in risky social behaviors. Study 1 will examine whether self-uncertainty predicts realistic and symbolic threat perceptions more for people high than low in conservatism, and Study 2 will examine whether realistic and symbolic threat perceptions, in turn, are positively or negatively associated with risky social behaviors, and whether conservatism moderates the strength of these relationships. See Figure 1 for the conceptual model for Study 2.

## Study 1

Data for Study 1 were collected in June 2020 using Amazon’s Mechanical Turk (MTurk), and examined whether conservatism moderated the relationship between self-uncertainty and perceptions of realistic and symbolic COVID-19 threat. In June 2020, the pandemic was raging, and its consequences were severe. As of June 28, 2020, the US had recorded almost 2.5 million confirmed cases and 124,811 deaths (World Health Organization, 2020). The United States had entered its first recession since 2009. Donald Trump had declined to reinstate federal lockdown orders, and he stated during a rally in Ohio that he wanted health authorities to decrease COVID-19 testing. Despite these claims, scientists and healthcare experts braced for a second wave of COVID-19 and warned that the pandemic was spiraling out of control (Phillip, 2020). This particular context of uncertainty and polarized beliefs surrounding the pandemic inspired our proposed model predicting polarized threat perceptions from self-uncertainty while considering Americans’ conservatism. We hypothesized that self-uncertainty should predict greater symbolic and realistic COVID-19 threat perceptions, and that the relationship between self-uncertainty and symbolic threat perceptions should be stronger for people higher (vs. lower) in conservatism. These data were part of a larger project, thus only the relevant measures are included in the procedure. The full project materials and data (for both studies) can be found on the project’s OSF page (https://osf.io/zuge4/?view_only=03f7c28b2e0549f4aaf00c02b72e9d24) .

### Method

Our sample included 315 participants: 175 men, 122 women, one nonbinary; average age of 37.03 (*SD* = 11.05); 187 White, 78 Black, 16 Hispanic/Latino, 34 other. This study was a correlational design where all measures were self-reported. Table 1 shows descriptive statistics for main study variables, and Tables 2 and 3 show the correlations between conservatism and all realistic and symbolic threat items.

**Table 1.** Reliabilities, means, standard deviations, and intercorrelations for main study variables: Study 1.

Variable	α	*M* (*SD*)	1	2	3	4
1. Self-uncertainty	.81	5.06 (1.28)	-	-	-	-
2. Conservatism	.88	5.00 (1.49)	.35***	-	-	-
3. Realistic threat	.83	5.05 (1.20)	.60***	.32***	-	-
4. Symbolic threat	.91	4.75 (1.48)	.47***	.49***	.74***	-

*Note*. ***p < .001.

**Table 2.** Correlations between the composite conservatism variable and all realistic threat items: Study 1.

Item	1	2	3	4	5
1. Conservatism	-	-	-	-	-
2. Your personal physical health	.28***	-	-	-	-
3. The physical health of citizens of the United States	.16**	.60***	-	-	-
4. Your personal financial safety	.33***	.49***	.46***	-	-
5. The U.S. economy	.22**	.39***	.59***	.42***	-
6. Day-to-day life for members of your local community	.36***	.52***	.57***	.48*	.53***

Note. *p < .05.**p < .01. ***p < .001.

**Table 3.** Correlations between the composite conservatism variable and all symbolic threat items: Study 1.

Item	1	2	3	4	5
1. Conservatism	-	-	-	-	-
2. The rights and freedoms of citizens of the United States	.48***	-	-	-	-
3. The values and traditions of citizens of the United States	.47***	.70***	-	-	-
4. What it means to be a citizen of the United States	.40***	.60***	.68***	-	-
5. The maintenance of law and order in the United States	.45***	.66***	.71***	.67***	-
6. American democracy	.42***	.62***	.63***	.57***	.66***

Note. ***p < .001.

#### Measures

##### Symbolic threat

Participants responded to a prompt asking, “To what extent do you consider the coronavirus outbreak a threat to. . .” by rating five symbolic threat sources (α = .91; 1 = *not a threat*, 7 = *extremely threatening*; adapted from Kachanoff et al., 2021). The symbolic threat sources included, “The rights and freedoms of citizens in the United States,” “The values and traditions of citizens of the United States,” “What it means to be a citizen of the United States,” “The maintenance of law and order in the United States,” and “American Democracy.”

##### Realistic threat

Participants responded to the same prompt for the Symbolic Threat Scale and rated the threat magnitude of five sources of realistic threat (α = .83; 1 = *not a threat*, 7 = *extremely threatening*; adapted from Kachanoff et al., 2021). The realistic threat sources included, “Your personal physical health,” “The physical health of citizens of the United States,” “Your personal financial safety,” “The U.S. economy,” and “Day to day life for members of your local community.”

##### Conservatism

A three-item scale (α = .88; 1 = *very liberal*, 7 = *very conservative*) asked participants, “How would you describe your [general political views/political views on social issues/political views on fiscal (monetary) issues]?”

##### Self-uncertainty

A five-item scale (adapted from Rast et al., 2012; α = .81; 1 = *strongly disagree*, 7 =  *strongly agree*) asked participants to respond to statements in relation to “how you feel about yourself and your future.” The items included, “I am uncertain about myself and the future,” “I am worried about myself and the future,” “I am concerned about myself and the future,” “At this very moment, I feel uncertain about myself,” and “At this very moment, I am uncertain about the future of the US.”

### Results

We conducted a sensitivity analysis using the “pwr2ppl” package in R (Aberson, 2022; R Core Team, 2023), finding at least 80% power to detect Δ*R*², comparable to some previous research (Jost et al., 2007; Kachanoff et al., 2021). See page 3 in the supplemental material for the detailed results of the sensitivity analyses.

#### Symbolic and realistic threat

Data were analyzed using hierarchical regression in R (R Core Team, 2023). Following recommendations from Aiken and West (1991), we mean-centered all predictor variables and used simple slopes tests to probe significant interactions. To test our hypotheses, we conducted a linear mixed effects model using “nlme” in R (Pinheiro et al., 2022), with self-uncertainty, conservatism, and threat type (realistic or symbolic) as the independent variables, with a random intercept by participant to account for the fact that each participant provided two threat observations. The dependent variable was perceived threat magnitude.

There was a main effect for all independent variables. Self-uncertainty (*b* = 0.40, *SE* = 0.05, *t* = 8.12, *p* < .001, 95% CI [0.30, 0.50]) and conservatism, (*b* = 0.38, *SE* = 0.04, *t* = 9.18, *p* < .001, 95% CI [0.30, 0.46]) positively predicted perceived threat magnitude, and participants perceived greater threat intensity for realistic than symbolic threat (*b* = 0.32, *SE* = 0.05, *t* = 5.91, *p* < .001, 95% CI [0.22, 0.43]). The significant three-way interaction between self-uncertainty, ideology, and threat type on perceived threat magnitude indicates that there was a significant difference in how conservatism and self-uncertainty interacted on perceived threat magnitude when the threat was symbolic compared to realistic (*b* = −0.06, *SE* = 0.02, *t* = −2.65, *p* = .008, 95% CI [−0.11, −0.02]). The interaction between self-uncertainty and conservatism on perceived threat magnitude was stronger for symbolic threat (*b* = 0.20, *SE* = 0.03, *t* = 7.76, *p* < .001, 95% CI [0.15, 0.25]) than realistic threat (*b* = 0.14, *SE* = 0.03, *t* = 5.35, *p* < .001, 95% CI [0.03, 0.19]).

**Figure 2.**
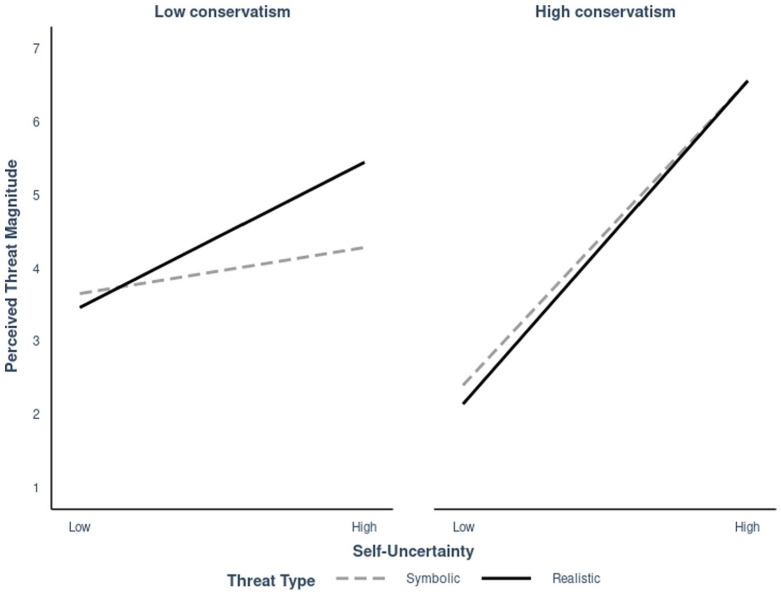
Threat magnitude predicted by self-uncertainty, moderated by threat type and conservatism in Study 1.

For people low in conservatism, self-uncertainty did not significantly predict perceived symbolic threat magnitude (*b* = 0.11, *SE* = 0.06, *t* = 1.72, *p* = .089, 95% CI [−0.02, 0.23]). However, self-uncertainty did predict realistic threat magnitude (*b* = 0.41, *SE* = 0.07, *t* = 6.32, *p* < .001, 95% CI [0.29, 0.54]). For people high in conservatism, symbolic threat magnitude (*b* = 0.69, *SE* = 0.06, *t* = 11.29, *p* < .001, 95% CI [0.57, 0.82]) and realistic threat magnitude (*b* = 0.74, *SE* = 0.06, *t* = 11.75, *p* ⩽ .001, 95% CI [0.61, 0.86]) were both positively associated with self-uncertainty (see Figure 2).

### Discussion

Consistent with our expectations, self-uncertainty predicted realistic threat among people low and high in conservatism, and it was a stronger predictor of symbolic threat among those high (vs. low) in conservatism. There was no statistically significant relationship between self-uncertainty and symbolic threat for people low in conservatism. Interestingly, conservatism was significantly positively correlated with all threat items, including threat to personal physical health and the physical health of American citizens. These findings provide support for the predictions derived from uncertainty-identity theory (Hogg, 2007, 2020, 2021, 2022), and are consistent with the different motivational underpinnings underlying conservative and liberal ideologies that are hypothesized by the motivated social cognition perspective (Jost & Amodio, 2012; Jost et al., 2003, 2007, 2008). Study 1 presents evidence that people high in conservatism perceived more realistic and symbolic threats as self-uncertainty increased, while people low in conservatism perceived more realistic threats, which are consistent with the threat types emphasized by liberal group norms. These findings pose an interesting question: if self-uncertain people high in conservatism perceive high levels of realistic and symbolic threat, which have competing relationships with adherence to public health measures (e.g., Chen et al., 2022; Kachanoff et al., 2021), what actions do they take with respect to virus spreading behaviors? Study 2 extended these findings by adding frequency of engagement in risky social behaviors as an outcome variable to examine this research question.

## Study 2

Data were collected in March 2021 via MTurk. At this time, 16% of the U.S. population had received one COVID-19 vaccination dose. President Biden had pledged to administer as many vaccine doses as possible during his first 100 days in office. The COVID-19 death toll had reached over half a million U.S. residents, but there was an overall decline in hospitalizations and deaths, and schools were beginning to reopen in some states for in-person instruction (Centers for Disease Control and Prevention, 2021). People were anticipating a “return to normal” now that vaccinations were becoming available, but this “new normal” would still look drastically different from prepandemic life for many people (e.g., Pinsker, 2021). For the first part of the study, participants responded to the same self-uncertainty, realistic threat, and symbolic threat measures from Study 1. Then, participants indicated the frequency with which they engaged in nine social behaviors that involved a heightened risk of viral transmission within the last 6 weeks. The full project materials can be found on the project’s OSF page (https://osf.io/zuge4/?view_only=03f7c28b2e0549f4aaf00c02b72e9d24).

As part of an exploratory project, we collected data on specific sources of realistic and symbolic threats, including how realistically and symbolically participants were threatened by public health guidelines, business closures and event cancellations, other American citizens not following public health guidelines, people exaggerating the negative impact of COVID-19, the Trump and Biden administrations’ management of the pandemic, powerful people using the virus as a strategy to profit off of American citizens, and wearing a mask. Descriptive statistics, correlations, and relationships to general realistic and symbolic threat measures can be found in the supplemental material (pp. 5–8).

### Method

#### Participants and design

Our sample included 361 participants: 216 men, 142 women; average age of 21.94 (*SD* = 11.32); 256 White, 54 Black, 25 Asian American, 26 other. This study was a correlational design where all measures were self-reported.

#### Procedure

Participants completed the same measures of self-uncertainty, conservatism, realistic threat, and symbolic threat as in Study 1, and then self-reported the frequency with which they had engaged in virus transmitting behaviors during the last 6 weeks. Table 4 contains descriptive statistics for all main study variables, and Tables 5 and 6 contain correlations between conservatism and all realistic and symbolic threat items.

**Table 4.** Reliabilities, means, standard deviations, and intercorrelations for main study variables: Study 2.

Variable	α	*M* (*SD*)	1	2	3	4
1. Self-uncertainty	.89	4.37 (1.84)	-	-	-	-
2. Conservatism	.93	4.11 (1.84)	.30***	-	-	-
3. Realistic threat	.85	4.60 (1.41)	.50***	.18***	-	-
4. Symbolic threat	.94	3.72 (1.93)	.42***	.48***	.54***	-
5. Social behaviors	.88	3.53 (1.98)	.47***	.50***	.29***	.63***

*** *p* < .001.

**Table 5.** Correlations between the composite conservatism variable and all realistic threat items: Study 2.

Item	1	2	3	4	5
1. Conservatism	-	-	-	-	-
2. Your personal physical health	.15*	-	-	-	-
3. The physical health of citizens of the United States	.02	.71***	-	-	-
4. Your personal financial safety	.24***	.54***	.48***	-	-
5. The U.S. economy	.17***	.45***	.54***	.53***	-
6. Day-to-day life in your local community	.17***	.69***	.66***	.61***	.58***

Note. *p < .05. ***p < .001.

**Table 6.** Correlations between the composite conservatism variable and all symbolic threat items: Study 2.

Item	1	2	3	4	5
1. Conservatism	-	-	-	-	-
2. Rights and freedoms of citizens of the United States	.42***	-	-	-	-
3. What it means to be an American	.41***	.77***	-	-	-
4. American values and traditions	.45***	.76***	.85***	-	-
5. American democracy	.46***	.74***	.81***	.85***	-
6. Maintenance of law and order in America	.43***	.70***	.77***	.81***	.86***

*Note*. ***p < .001.

##### Risky social behaviors

Participants reported the frequency with which they engaged in 15 behaviors in the last 6 weeks. All items used the following response scale: 1 = “*never*,” 2 = “*rarely* (in less than 10% of the chances you have had),” 3 = “*occasionally* (in about 30% of the chances you have had),” “*sometimes* (in about 50% of the chances you have had),” 4 =“*frequently* (in about 70% of the chances you have had),” 5 =“*usually* (in about 90% of the chances you have had),” 6 =“*always* (100% of the chances you have had),” 7 =“I have not had any chances to do this.” The behaviors included attendance at nonessential social gatherings, attendance at crowded places (e.g., parties, concerts, sports games, festivals), engagement in nonessential travel, attendance at a dining establishment and consumption of food or drinks inside the establishment, visitation of family with/without social distancing, visiting friends with/without social distancing, engagement in common close contact greetings maintenance of a 6 foot distance between themselves and others, mask wearing, and hand washing and sanitizing. We selected all items based on research that identified them as risky (e.g., Kim & Crimmins, 2020; Salimi et al., 2020) and physician assessments of which behaviors presented the most risk of spreading the virus (e.g., Infectious Diseases Society of America, n.d.; Texas Medical Association, 2020).

As our research pertains to risky social behaviors, we excluded the items that measured basic hygiene behaviors (keep a 6-foot difference between self and others, wear a mask around others, sanitize hands). We also removed the items that measured frequency of socializing with friends/family/vulnerable populations while engaging in social distancing measures to include only behaviors with the highest risk of virus transmission. The final measure included nine items asking the frequency of attending nonessential social gatherings, attending crowded events, engaging in nonessential travel, dining inside and outside at a bar/restaurant/café, visiting family/friends/vulnerable populations without social distancing, and common close contact greetings.

### Results

We conducted a sensitivity analysis using the “pwr2ppl” package in R (Aberson, 2022; R Core Team, 2023), finding at least 80% power to detect correlations between main study variables that are comparable to some previous research (Jost et al., 2007; Kachanoff et al., 2021). See page 6 in the supplemental material for the detailed results of the sensitivity analysis.

#### Risky social behaviors

**Figure 3.**
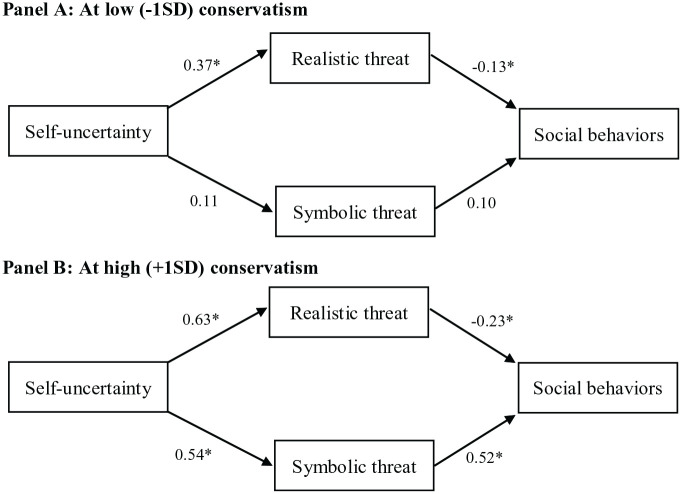
Indirect effect of self-uncertainty on risky social behaviors through threat perception moderated by conservatism in Study 2. Panel A: At low (−1 *SD*) conservatism. Panel B: At high (+1 *SD*) conservatism. *Note.* Panels A and B represent the association with self-uncertainty for low and high conservatism, respectively, in a model that includes the interaction between self-uncertainty and conservatism onto the mediators, and an interaction between self-uncertainty and conservatism onto risky social behaviors. *confidence interval does not include zero.

We conducted a parallel moderated mediation using lavaan with symbolic and realistic threats entered simultaneously as mediators (R Core Team, 2023; Rosseel, 2012). In other words, self-uncertainty, conservatism, and their interaction served as predictors of realistic threat, symbolic threat, and social behavior; realistic threat and symbolic threat also served as predictors of social behavior. See Figure 3 for path coefficients; see Tables 7 and 8 for complete statistical reporting.

**Table 7.** Study 2: Test of conditional indirect effects of self-uncertainty on risky social behaviors, through symbolic threat and moderated by conservatism.

	*b* (*SE*)	*z*	95% CI
**Outcome: Symbolic threat**			
Self-uncertainty	0.33* (0.05)	6.53	[0.23, 0.42]
Conservatism	0.35* (0.05)	6.91	[0.25, 0.45]
Interaction term	0.22* (0.05)	4.73	[0.12, 0.31]
**Simple slopes: Symbolic threat**			
Low conservatism	0.11 (0.07)	0.14	[−0.04, 0.24]
High conservatism	0.54* (0.07)	8.32	[0.42, 0.68]
**Outcome: Behaviors**			
Self-uncertainty	0.58* (0.10)	5.82	[0.36, 0.75]
Conservatism	0.46* (0.10)	4.80	[0.28, 0.66]
Symbolic threat	0.96* (0.11)	8.38	[0.74, 1.19]
Interaction term	0.19* (0.08)	2.31	[0.03, 0.36]
**Conditional indirect effects: Symbolic threat**			
Low conservatism	0.10 (0.07)	1.43	[−0.03, 0.24]
High conservatism	0.52* (0.09)	5.64	[0.34, 0.71]

*Note.* Interaction term is Self-Uncertainty x Conservatism. Parameter estimates calculated with 1,000 bootstrapped iterations.

* indicates statistical significance.

**Table 8.** Test of conditional indirect effects of self-uncertainty on risky social behaviors, through realistic threat and moderated by conservatism: Study 2.

	*b* (*SE*)	*z*	95% CI
**Outcome: Realistic threat**			
Self-uncertainty	0.50* (0.06)	8.99	[0.40, 0.61]
Conservatism	0.02 (0.05)	0.47	[−0.08, 0.11]
Self-Uncertainty x Conservatism	0.13* (0.05)	2.69	[0.03, 0.23]
**Simple slopes: Realistic threat**			
Low conservatism	0.37* (0.08)	4.93	[0.23, 0.52]
High conservatism	0.63* (0.07)	8.60	[0.48, 0.77]
**Outcome: Behaviors**			
Self-uncertainty	0.58* (0.10)	5.82	[0.36, 0.75]
Conservatism	0.46* (0.10)	4.80	[0.28, 0.66]
Realistic threat	−0.36* (0.10)	−3.64	[−0.55, −0.02]
Self-Uncertainty x Conservatism	0.19* (0.08)	2.31	[0.03, 0.36]
**Conditional indirect effects: Realistic threat**			
Low conservatism	−0.13* (0.05)	−2.91	[−0.24, −0.06]
High conservatism	−0.23* (0.07)	−3.22	[−0.37, −0.09]

*Note.* Interaction term is Self-Uncertainty x Conservatism. Parameter estimates calculated with 1,000 bootstrapped iterations.

* indicates statistical significance.

The index of moderated mediation was significant for realistic threat (index = −0.05, *SE* = 0.02, 95% CI [−0.11, −0.01]) and symbolic threat (index = 0.21, *SE* = 0.05, 95% CI [0.11, 0.31]). Realistic threat partially mediated the relationship between self-uncertainty and risky social behaviors for people low and high in conservatism. 12 conservatism, self-uncertainty was associated with higher levels of risky social behaviors through symbolic threat.

### Discussion

The results of Study 2 indicate that conservatism moderates the relationship between self-uncertainty and threat perception in the same manner as in Study 1, and, in turn, realistic and symbolic threat perceptions have different relationships with frequency of engagement in risky social behaviors. For people high and low in conservatism, self-uncertainty predicted realistic threat, which generally had a modest negative association with frequency of engagement in risky social behaviors. Only self-uncertain people high in conservatism perceived more symbolic threat, which, in turn, predicted greater frequency of engagement in risky social behaviors. The positive association between symbolic threat and risky social behaviors appeared stronger than the negative association between realistic threat and risky social behaviors, although we did not formally test this claim. This provides some explanation for why self-uncertain conservatives may be most likely to perceive realistic and symbolic threat, but conservatives overall engage in less public health preserving behaviors than liberals (Clinton et al., 2021; Gollwitzer et al., 2020; Latkin et al., 2022). In other words, people who perceive high levels of both types of threat (i.e., highly self-uncertain people high in conservatism) may still engage in more risky social behaviors than those low in symbolic threat (i.e., liberals) because the negative association between realistic threat and behaviors may be counteracted by the positive association between symbolic threat and behaviors. This theoretical proposition should be experimentally tested before reaching definite conclusions, but it allows for a more nuanced understanding of the complex relationships between threat perception and engagement in virus spreading behaviors in the COVID-19 context.

The results of Study 2 are consistent with the predictions of uncertainty-identity theory (Hogg, 2007, 2020, 2021, 2022), which posits that group norms should dictate those aspects of the pandemic that are threatening and prescribe a group normative manner to respond to the threats. Although the motivated social cognition framework (Jost & Amodio, 2012; Jost et al., 2003, 2007, 2008) predicts that self-uncertain conservatives should perceive greater levels of both realistic and symbolic threat, conservative norms emphasized symbolic pandemic threats, such as threats to individual freedoms (Collins et al., 2021; Jost, 2017; Jost et al., 2003; Kempthorne & Terrizzi, 2021; Kerr et al., 2021; van Kessel & Quinn, 2020; Waytz et al., 2019), which, in turn, had a strong association with risky social behaviors. For people low in conservatism, self-uncertainty was associated with realistic threats but not with symbolic threats, and, accordingly, it predicted less engagement in risky social behaviors. Our findings also provide support for the motivated social cognition perspective (Jost & Amodio, 2012; Jost et al., 2003, 2007, 2008), in that self-uncertainty predicted more realistic and symbolic threat among people high in conservatism, and conservatism was positively correlated with all realistic and symbolic threat items except for perceived threat to the physical health of all Americans.

## General Discussion

Across two correlational studies, self-uncertainty was positively associated with realistic and symbolic threat perception for people high in conservatism, but only associated with realistic threat perception for people low in conservatism. For people high and low in conservatism, self-uncertainty was associated with realistic threat perceptions, which were associated with lower levels of engagement in risky social behaviors. For those high in conservatism, self-uncertainty was also associated with symbolic threat perceptions, which were associated with higher levels of engagement in risky social behaviors. These findings support the body of literature showing that self-uncertainty ties perceptions and behaviors to group norms (Choi & Hogg, 2020; Grant & Hogg, 2012; Grieve & Hogg, 1999; Hogg & Mahajan, 2018; Hogg et al., 1998, 2010, 2011; Hohman & Hogg, 2015; Mullin & Hogg, 1999; Smith & Hogg, 2008), and extend this into group-norms-based COVID-19 threat perception and corresponding behaviors.

Interestingly, conservatism was positively correlated with all threat items in Study 1, and with all threat items except the physical health of Americans in Study 2. Moreover, people high in conservatism exhibited a stronger association between self-uncertainty and threat perceptions. Conservatism was associated with physical, economic, and meaning-related threat items in both studies, so self-uncertainty may have amplified existing threat perception amongst those high in conservatism (if the relationships observed are partially causal). This points to an integration between uncertainty-identity theory and the motivated social cognition framework—self-uncertainty predicted group normative threat perception for both people high and low in conservatism and seemed to emphasize threat perception overall for people high in conservatism.

Our theoretical model predicted that self-uncertain conservatives should be the most realistically and symbolically threatened, which was supported by Studies 1 and 2. These findings are juxtaposed against research that found realistic threat perception, including perceived threat or susceptibility to the virus, is associated with liberalism, not conservatism (Kachanoff et al., 2021; Kempthorne & Terrizzi, 2021; Kerr et al., 2021). It is possible that self-uncertainty, in addition to promoting adherence to group-norms-based perceptions, also affects threat perception for people high in conservatism in a broader sense. Study 2 extends these findings by demonstrating that the self-reported frequency of risky social behaviors amongst highly self-uncertain people high in conservatism is associated with threat type, and the stronger positive relationship between symbolic threat and behaviors may somewhat counteract the weaker negative relationship between realistic threat and behaviors. Thus, the lower frequency of public health preserving behaviors reported amongst conservatives may be more nuanced than it appears on the surface.

### Limitations and Future Research Directions

As these data are correlational, there is a clear need for experimental replications and extensions to properly test the theoretical model we have put forth. For example, manipulating self-uncertainty and group identity salience and measuring pro- and antinormative threat perception in a non-COVID-19 domain would provide more information as to whether liberals and conservatives exhibit different reactions to self-uncertainty. Such an experiment would also provide a test of whether self-uncertainty simultaneously maximizes group normative threat perception while minimizing threat perception that is not prescribed by group norms (Hogg et al., 1990; Turner et al., 1987). In our sample, it is very likely that COVID-19-related threats were at least part of the reason that some participants indicated uncertainty about “the future of the US” (one of the uncertainty items), suggesting that some of our observed relationships were causally reversed. Also, we cannot rule out correlations due to unmeasured variables such as misinformation, which could contribute to both uncertainty and threats.

In addition, we did not collect a sample that was demographically representative of the United States population, and doing so would have increased the generalizability of our results. Collecting data only in the United States means that these findings may not generalize to political ideology globally. Finally, our risky social behaviors measure was limited in that it relied on self-report of behaviors in the last 6 weeks. This introduces the potential for variance in the accuracy of self-reported behaviors reflecting actual behaviors.

## Concluding Remarks

COVID-19 incited large-scale system instability in the U.S. that undermined people’s understanding of themselves and their ability to predict their world, which shifted attention to a relevant group membership (i.e., political ideology) that provided normative frameworks for deciding which aspects of the pandemic to focus attention on and how to appropriately respond. The findings presented in this paper are consistent with uncertainty-identity theory’s (Hogg, 2007, 2020, 2021, 2022) hypothesis that self-uncertainty ties people’s perceptions and behaviors to a salient group membership, for example, political ideology. These findings are also consistent with the motivated social cognition framework (Jost & Amodio, 2012; Jost et al., 2003, 2007, 2008), and suggest that self-uncertainty may emphasize a broader range of threat perceptions for people high in conservatism. Large-scale external events invoke many types of uncertainties and threats, and future research directions can examine whether self-uncertainty causes group normative threat perception and corresponding behaviors for liberals and conservatives in a COVID-19 context or otherwise.

## Supplemental Material

sj-docx-1-gpi-10.1177_13684302231180525 – Supplemental material for Self-uncertainty and conservatism during the COVID-19 pandemic predict perceived threat and engagement in risky social behaviors Click here for additional data file. Supplemental material, sj-docx-1-gpi-10.1177_13684302231180525 for Self-uncertainty and conservatism during the COVID-19 pandemic predict perceived threat and engagement in risky social behaviors by Lily Syfers, Alexandria Jaurique, Benjamin Anjierwerden, Sara E. Burke, Justin D. Hackett, David E. Rast and Amber M. Gaffney in Group Processes & Intergroup Relations
