# Supplementary material for: Self-uncertainty and conservatism during the COVID-19 pandemic predict perceived threat and engagement in risky social behaviors
Source: Group Process Intergroup Relat. 2023 Jul 7:13684302231180525. doi: 10.1177/13684302231180525 (PMC10331121; doi:10.1177/13684302231180525)
Supplement: sj-docx-1-gpi-10.1177_13684302231180525 – Supplemental material for Self-uncertainty and conservatism during the COVID-19 pandemic predict perceived threat and engagement in risky social behaviors [file sj-docx-1-gpi-10.1177_13684302231180525.docx]

Table of Contents

[Study 1 2](#_Toc126167641)

[Background variables for study 1 2](#_Toc126167642)

[Table S1 3](#_Toc126167643)

[Sensitivity Effect Size Analysis Parameters for Study 1 3](#_Toc126167644)

[Study 2 4](#_Toc126167645)

[Background variables for study 2 4](#_Toc126167646)

[Table S2 5](#_Toc126167647)

[Parameters used in effect sensitivity analysis for two-way regression interactions for Study 2 5](#_Toc126167648)

[Table S3 6](#_Toc126167649)

[Parameters used in effect sensitivity power analysis for moderated mediation in Study 2 6](#_Toc126167650)

[Table S4 7](#_Toc126167651)

[Confirmatory factor analysis for study 2 risky social behavior measure 7](#_Toc126167652)

[Table S5 8](#_Toc126167653)

[Descriptive statistics for specific realistic and symbolic threat sources 8](#_Toc126167654)

[Table S6 9](#_Toc126167655)

[Specific threat sources predicting general symbolic threat and general realistic threat 9](#_Toc126167656)

[Table S7 10](#_Toc126167657)

[Correlations between realistic threat sources and key study variables 10](#_Toc126167658)

[Table S8 11](#_Toc126167659)

[Correlations between symbolic threat sources and key study variables 11](#_Toc126167660)

# Study 1

## Background variables for study 1

The regression of age, gender, income, race and education showed only two significant effects on either of the dependent variables. Gender significantly predicted symbolic threat, *b** = -.19, *t* = -3.39, *p* = .001, 95% C.I. [-0.30, -0.08], and race significantly predicted realistic threat, *b** = -.17, *t* = -3.06, *p* = .002, 95% C.I. [-.29, -.06]. Including gender and race as covariates in the hypothesized two-way interactions did not change the significant pattern of results, so we present analyses without gender and race as covariates.

## Table S1

### Sensitivity Effect Size Analysis Parameters for Study 1

| *R²* model = .30 | *N* = 315 |
| --- | --- |
| Δ*R²* | Power |
| .018 | 80% |
| .023 | 90% |
| .03 | 95% |

# Study 2

## Background variables for study 2

Age significantly predicted both symbolic threat, *b** = -.12, *t* = -2.29, *p* = .023, 95% C.I. [-0.22, -0.02] and realistic threat, *b** = -.13, *t* = -2.36, *p* = .019, 95% C.I. [-0.23, -0.02]. Gender significantly predicted symbolic threat, *b** = 0.14, *t* = 2.64, *p* = .009, 95% C.I. [0.04, 0.25] but did not predict realistic threat, *b** = -0.05, *t* = -0.89, *p* = .370, 95% C.I. [-0.16, 0.06]. Education significantly predicted symbolic threat, *b** = 0.16, *t* = 3.11, *p* = .002, 95% C.I. [0.06, 0.27] and realistic threat, *b** = 0.15, *t* = 2.86, *p* = .005, 95% C.I. [0.05, 0.25]. Race did not significantly predict symbolic threat, *b** = -.04, *t* = -0.69, *p* = .486, 95% C.I. [-0.14, 0.07] or realistic threat, *b** = -.002, *t* = -0.04, *p* = .965, 95% C.I. [-0.04, 0.10].

## Table S2

### Parameters used in effect sensitivity analysis for two-way regression interactions for Study 2

| *R²* model = .30 | *N* = 361 |
| --- | --- |
| Δ*R²* | Power |
| .016 | 80% |
| .02 | 90% |
| .025 | 95% |

## Table S3

### Parameters used in effect sensitivity power analysis for moderated mediation in Study 2

| Parameter | 80% power | 90% power | 95% power |
| --- | --- | --- | --- |
| Self-uncertainty, social behaviors | *r* = .10 | *r* = .10 | *r* = .10 |
| Self-uncertainty, symbolic threat | *r* = .19 | *r* = .21 | *r* = .22 |
| Self-uncertainty, conservatism | *r* = .10 | *r* = .10 | *r* = .10 |
| Self-uncertainty, interaction | *r* = 0 | *r* = 0 | *r* = 0 |
| Symbolic threat, social behaviors | *r* = .19 | *r* = .21 | *r* = .23 |
| Conservatism, social behaviors | *r* = .19 | *r* = .21 | *r* = .22 |
| Interaction, social behaviors | *r* = 0 | *r* = 0 | *r* = 0 |
| Conservatism, interaction | *r* = 0 | *r* = 0 | *r* = 0 |
| Symbolic threat, interaction | *r* = .19 | *r* = .21 | *r* = .23 |
| Symbolic threat, conservatism | *r* = .10 | *r* = .10 | *r* = .10 |

*Note*. Sensitivity calculated with 1,000 simulations. Person’s *r* is reported.

## Table S4

### Confirmatory factor analysis for study 2 risky social behavior measure

| Model | χ^2^(*df*) | Δ χ^2^(*df*) | *p* | TLI | CFI | SRMR |
| --- | --- | --- | --- | --- | --- | --- |
| Model 1 | 584.51(54) |  |  | .86 | .88 | .11 |
| Model 2 | 186.34(53) | 398.17(1) | < .001 | .97 | .97 | .03 |

*Note.* Model 1 is all items loaded onto one factor. Model 2 is two factors. Factor 1 includes is three items measuring frequency of hand sanitizing, keeping six-foot distance from others, wearing a mask around others. Factor 2 includes 9 items measuring frequency of attending non-essential social gatherings, attending crowded places (e.g., parties, concerts, sports games, festivals), engaging in non-essential travel, visiting a dining establishment and consuming food or drinks inside or outside the establishment, visiting family and did *not* social distance, visiting friends and did *not* social distance, visited a population vulnerable to COVID-19 and did *not* social distance, engaged in common close contact greetings (e.g., hugs, handshakes, kissing).

## Table S5

### Descriptive statistics for specific realistic and symbolic threat sources

|  |  | |  | |
| --- | --- | --- | --- | --- |
| **Source of Threat** | Symbolic Threat | | Realistic Threat | |
|  | Mean (*SD*) | $\alpha$ | Mean (*SD*) | $\alpha$ |
| COVID-19 public health guidelines | 3.59 (1.99) | .93 | 3.96(1.82) | .91 |
| Business closures and event cancellations | 3.66(1.95) | .93 | 3.86(1.72) | .88 |
| American citizens who do not follow COVID-19 public health guidelines | 3.94(1.87) | .92 | 4.22(1.73) | .89 |
| Trump administration’s management of the COVID-19 pandemic | 4.07(1.91) | .93 | 4.27(1.82) | .92 |
| Being infected with the COVID-19 virus | 3.41(1.98) | .93 | 4.67(1.70) | .87 |
| Biden administration’s management of the COVID-19 pandemic | 3.46(2.05) | .95 | 3.56(1.94) | .94 |
| American citizens exaggerating the negative impact of the virus | 3.84(1.88) | .93 | 3.93(1.80) | .91 |
| Powerful people in America (e.g., politicians, wealthy people, celebrities) using the virus as a strategy to profit off American citizens | 4.23(1.79) | .91 | 4.14(1.74) | .91 |
| Wearing a mask | 3.26(2.05) | .95 | 3.15(2.02) | .95 |

## Table S6

### Specific threat sources predicting general symbolic threat and general realistic threat

| **Threat Source** | *Outcome: General*  *Symbolic Threat* | | | *Outcome: General*  *Realistic Threat* | | | |
| --- | --- | --- | --- | --- | --- | --- | --- |
|  | *b*(*SE*) | *t* | *p* | | *b*(*SE*) | *t* | *p* |
| COVID-19 public health guidelines | 0.44(0.06) | 6.83 | <.001 | | 0.13(0.05) | 2.76 | .01 |
| Business closures and event cancellations | 0.22(0.07) | 3.19 | <.001 | | 0.12(0.06) | 1.94 | .05 |
| American citizens who do not follow COVID-19 public health guidelines | 0.03(0.05) | 0.57 | .57 | | 0.12(0.05) | 2.14 | .03 |
| Trump administration’s management of the COVID-19 pandemic | 0.01(0.04) | 0.20 | .84 | | 0.08(0.04) | 1.92 | .06 |
| Being infected with the COVID-19 virus | 0.22(0.04) | 4.95 | <.001 | | 0.29(0.05) | 6.12 | <.001 |
| Biden administration’s management of the COVID-19 pandemic | -0.03(0.05) | -0.61 | .54 | | 0.04(0.04) | 0.80 | .43 |
| American citizens exaggerating the negative impact of the virus | -0.03(0.04) | -0.72 | .47 | | -0.01(0.04) | -0.25 | .80 |
| Powerful people in America (e.g., politicians, wealthy people, celebrities) using the virus as a strategy to profit off American citizens | 0.00(0.04) | 0.09 | .93 | | 0.09(0.04) | 2.28 | .02 |
| Wearing a mask | 0.11(0.05) | 2.35 | .02 | | -0.07(0.04 | -1.62 | .11 |

## Table S7

| Variable | *M*(*SD*) | 1 | 2 | 3 | 4 | 5 | 6 | 7 |
| --- | --- | --- | --- | --- | --- | --- | --- | --- |
| Physical infection with the virus | 4.68(1.66) | - |  |  |  |  |  |  |
| Public health guidelines | 4.03(1.81) | 0.40* | - |  |  |  |  |  |
| American citizens who do not follow public health guidelines | 4.27(1.69) | 0.77* | 0.57* | - |  |  |  |  |
| Powerful people using the virus as a strategy for profit | 4.16(1.76) | 0.57* | 0.64* | 0.66* | - |  |  |  |
| Conservatism | 4.15(1.81) | 0.03 | 0.37* | 0.11* | 0.24* | - |  |  |
| Uncertainty | 4.24(1.57) | 0.39* | 0.46* | 0.48* | 0.41* | 0.30* | - |  |
| Risky social behaviors | 3.53(2.04) | 0.02 | 0.53* | 0.23* | 0.40* | 0.51* | 0.46* | - |

### Correlations between key realistic threat sources and key study variables

## Table S8

### Correlations between key symbolic threat sources and key study variables

| Variable | *M*(*SD*) | 1 | 2 | 3 | 4 | 5 | 6 | 7 |
| --- | --- | --- | --- | --- | --- | --- | --- | --- |
| Public health guidelines | 4.68(1.66) | - |  |  |  |  |  |  |
| Business and event closures | 4.03(1.81) | 0.92* | - |  |  |  |  |  |
| Mask wearing | 4.27(1.69) | 0.82* | 0.79* | - |  |  |  |  |
| Physical infection with the virus | 4.16(1.76) | 0.66* | 0.68* | 0.62* | - |  |  |  |
| Conservatism | 4.15(1.81) | 0.50* | 0.50* | 0.55* | 0.36* | - |  |  |
| Uncertainty | 4.24(1.57) | 0.40* | 0.38* | 0.41* | 0.47* | 0.30* | - |  |
| Risky social behaviors | 3.53(2.04) | 0.66* | 0.64* | 0.75* | 0.54* | 0.51* | 0.46* | - |
